# LmTraceMap: A *Listeria monocytogenes* fast-tracing platform for global surveillance

**DOI:** 10.1371/journal.pone.0267972

**Published:** 2022-05-09

**Authors:** Yen-Yi Liu, Chih-Chieh Chen, Chien-Hua Yang, Hui-Yi Hsieh, Jia-Xin He, Hao-Hsuan Lin, Chi-Ching Lee

**Affiliations:** 1 Department of Public Health, China Medical University, Taichung, Taiwan; 2 Institute of Medical Science and Technology, National Sun Yat-sen University, Kaohsiung, Taiwan; 3 Department of Computer Science and Information Engineering, Chang Gung University, Taoyuan, Taiwan; 4 Genomic Medicine Core Laboratory, Chang Gung Memorial Hospital, Taoyuan, Linkou, Taiwan; 5 Artificial Intelligence Research Center, Chang Gung University, Taoyuan, Taiwan; Zhejiang University, CHINA

## Abstract

*Listeria monocytogenes* can cause listeriosis, and people with hypoimmunity such as pregnant women, infants and fetuses are at high risk of invasive infection. Although the incidence of listeriosis is low, the fatality rate is high. Therefore, continual surveillance and rapid epidemiological investigation are crucial for addressing *L*. *monocytogenes*. Because of the popularity of next-generation sequencing, obtaining the whole-genome sequence of a bacterium is easy. Several genome-based typing methods are available, and core-genome multilocus sequence typing (cgMLST) is the most recognized methods. Using cgMLST typing to compare *L*. *monocytogenes* whole-genome sequences (WGS) with those obtained across distinct regions is beneficial. However, the concern is how to incorporate the powerful cgMLST method into investigations, such as by using source tracing. Herein, we present an easy-to-use web service called–**LmTraceMap** (http://lmtracemap.cgu.edu.tw/hua_map/test/upload.php; http://120.126.17.192/hua_map/test/upload.php) that can help public-health professionals rapidly trace closely related isolates worldwide and visually inspect them in search results on a world map with labeled epidemiological data. We expect the proposed service to improve the convenience of public health investigations.

## Introduction

Listeriosis is an infectious disease caused by *Listeria monocytogenes* [1]. The clinical outcomes vary depending on whether the infection is invasive or non-invasive [2]. The severity of listeriosis is usually related to the patients’ immunity [3]. The common symptoms of *L*. *monocytogenes* infection are diarrhea, nausea and gastrointestinal disorders [4]. However, some people with hypoimmunity, such as elderly patients, pregnant women, infants, and fetuses, are at high risk of invasive listeriosis [5–7]. Invasive listeriosis can result in sepsis, meningitis meningoencephalitis, and death in the hypoimmune population [8]. Although the incidence of listeriosis is low (0.34 in 100000 population), the fatality rate is high (~26%) compared with other food-borne diseases, according to the WHO 2015 statistic reports [9]. Listeriosis infections can not only cause a high fatality rate but also show to a long incubation period [10]. Historical outbreaks such as the 2018 outbreak in Australia [11, 12] caused 20 infected cases and 7 deaths from the infection.

The infectious agent of listeriosis is *L*. *monocytogenes*, which is a facultative anaerobic gram-positive bacterium that can be cultured from the environment [13]. *L*. *monocytogenes* can grow at low temperatures (e.g. -1.5°C) and is tolerant to low humidity and high-temperature (e.g. 72°C), acid, salt, and alcohol conditions [14–17]. Therefore, people are at high risk of acquiring the pathogen from ingestion of contaminated lettuce or ready-to-eat food [18]. *L*. *monocytogenes* can be classified into 14 serotypes according to O and H antigens [19], and 1/2a, 1/2b and 4b (the most observed type) are the primary types that can cause human listeriosis [20].

With the decreasing price of next-generation sequencing systems, public-health researchers are increasingly applying genomic epidemiology methods such as wgSNP [21], MLST [22], wgMLST [23], pMLST [24] and cgMLST [25] in surveillance and source tracking investigations. Additionally, PulseNet [26], which is one of the largest foodborne disease (including listeriosis) surveillance networks, has transitioned from a pulsed-field gel electrophoresis method to a whole genome sequencing (WGS) method for foodborne diseases investigation [27]. Therefore, comparing the *L*. *monocytogenes* WGS with sequencing systems from other countries is beneficial.

Herein, we propose a platform–**LmTraceMap** (http://lmtracemap.cgu.edu.tw/hua_map/test/upload.php) that can provide public-health researchers fast access to compare their *L*. *monocytogenes* WGS results with isolates from other countries to track potential sources. A large database containing > 20,000 *L*. *monocytogenes* WGS results was created (Lm trace database). The gene-by-gene comparison method [28] was applied for the genome comparison. On the platform, the tracking results are not only listed in a table format but also labeled on a map for convenient use.

## Methods and implementation

To construct the tracking platform, we downloaded 20,683 *L*. *monocytogenes* WGS from the NCBI SRA database (S1 File). The gene-by-gene comparison method was applied to calculate the genomic distance for the genetic relatedness analysis. The typing scheme used for the gene-by-gene comparison was the cgMLST defined by Ruppitsch et al. [29].

### Scheme used in cgMLST typing

The typing scheme comprise 1,701 loci. The alleles were downloaded from the Ridom allelic nomenclature server (https://www.cgmlst.org/ncs/schema/690488/). The allelic profiling (S2 File) was performed using the PGAdb-builder database creation tool [30].

### Description of *L*. *monocytogenes* WGS isolates downloaded from NCBI SRA database

The time-span of the downloaded isolates was 1921–2021, with most of the isolates collected between 2010 and 2021 (S1 Fig). In total, 283 ST types (S1 Table) were identified for the downloaded isolates with ST5 comprising the most isolates and ST1 comprising only one isolate. The downloaded isolates can also divided into 6 serogroups, namely, “1/2a, 3a”, “1/2b, 3b”, “1/2c, 3c”, “4b, 4d, 4e”, “4b, 4d, 4e*” and “nontypeable”, by using LisSero software prediction [31] (S2 Fig). The downloaded genomes were transformed into allelic profiles by using the cgMLST scheme, and the transformed profiles were stored in a flattened file. The epidemiological data for each of the downloaded genomes were stored in a MySQL database.

### Flowchart of building the LmTraceMap

**Fig 1:**
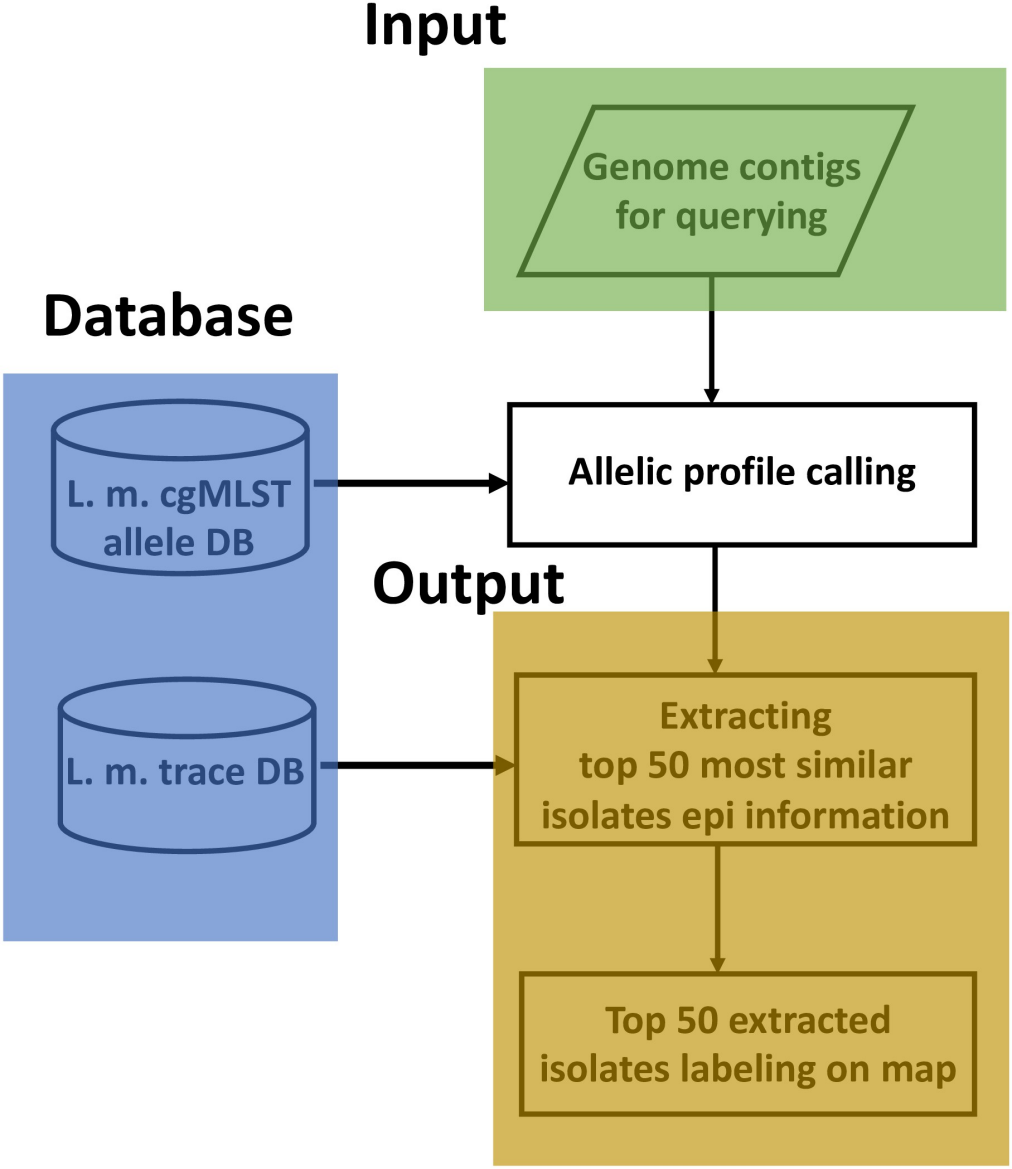
Schematic flowchart of LmTranceMap. System workflow primarily includes 3 parts, namely *Input*, *Output*, and *Database*, connected through “allelic profile calling”.

The flowchart of the LmTraceMap is presented in Fig 1. Three compartments, namely “Input”, “Output”, and “Database”, are labeled. A draft genome of *L*. *monocytogenes* must be uploaded into the LmTraceMap for the initial calculation. The uploaded draft genome is assembled by SPAdes [32] using the same parameters and subsequently transformed to an allelic profile through a Lm cgMLST-based allele database. The epidemiological data for the top 50 most similar isolates are extracted from the “Lm trace database”. The top 50 extracted isolates are subsequently labeled on the word map. The epidemiological data and labeled word map of the extracted top 50 isolates can be downloaded.

### Web server

The web server was constructed using PHP, Python, and Perl scripts and the MySQL database. The Bootstrap framework is used to render the result form downloadable. Leaflet (https://leafletjs.com/), an open source JavaScript library for constructing web-mapping applications, is used to visualize the tracking results. For email management, the Mailutils general-purpose mail package (https://mailutils.org) is used to automatically send the results through email to inform the user. For browsing the LmTraceMap, we recommend using Chrome, Safari and Firefox browsers to avoid the problem.

### Input format

Users can acquire a test file from our “Input/Output Sample” icon in the upper right corner of our web server to sample how LmTraceMap works. The genome assembly of *L*. *monocytogenes* (in .*fa*, .*fasta*, and .*fna* file extensions) is required as the input data for initializing the service. Only one genome at a time is available.

### The description of the web interface of LmTraceMap

**Fig 2:**
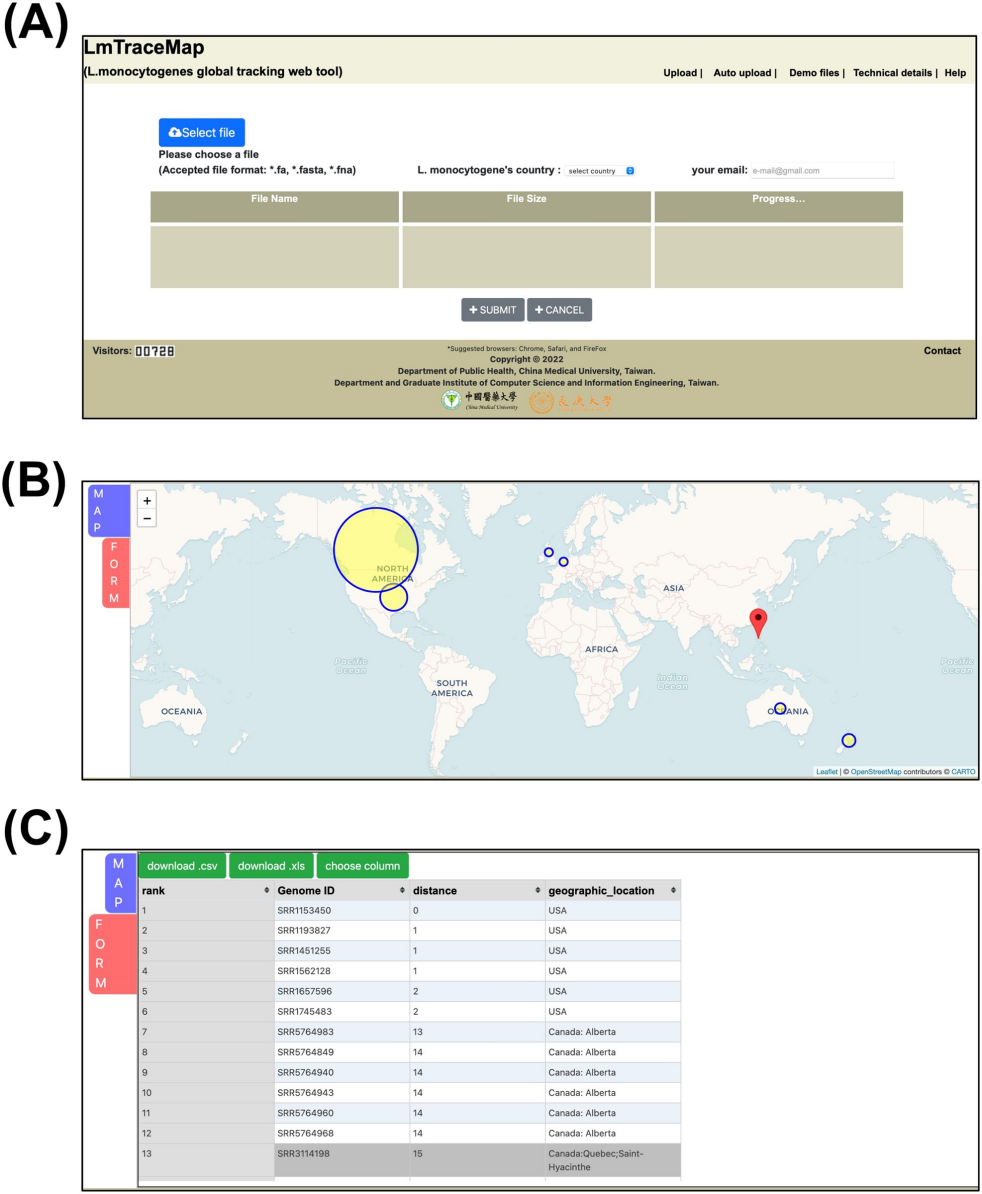
LmTraceMap screenshot. a) LmTraceMap server homepage; b) MAP tag of result page; c) FORM tag of result page. (Base map and data from OpenStreetMap and OpenStreetMap Foundation. Reprinted from OpenStreetMap under a CC BY license, with permission from OpenStreetMap, original copyright 2020. ©OpenStreetMap contributors. Annotated by the authors).

The home page of LmTraceMap is presented in Fig 2(a). Users must upload their query genome through the *select file* option. Country data must be selected from the pull-down list. A user’s email address is required to receive emailed results. The result page contains 2 tags: *MAP* (Fig 2(b)) and *FORM* (Fig 2(c)). On the *MAP* page, the nations with the top 50 most similar isolates are labeled on the word map with yellow circles sized according to the number of isolates. The yellow circles can be clicked with a mouse to display a list comprising all of the isolates within the circle. The location of the user- uploaded genome is also displayed as an inverted tear drop icon. On the *FORM* page, the epidemiological data of the top 50 isolates are presented as a table. All of the columns can be added and removed by selecting the *choose column* option. The final selected columns can be downloaded in.*csv* or.*xls* formats.

### Example analysis

**Fig 3:**
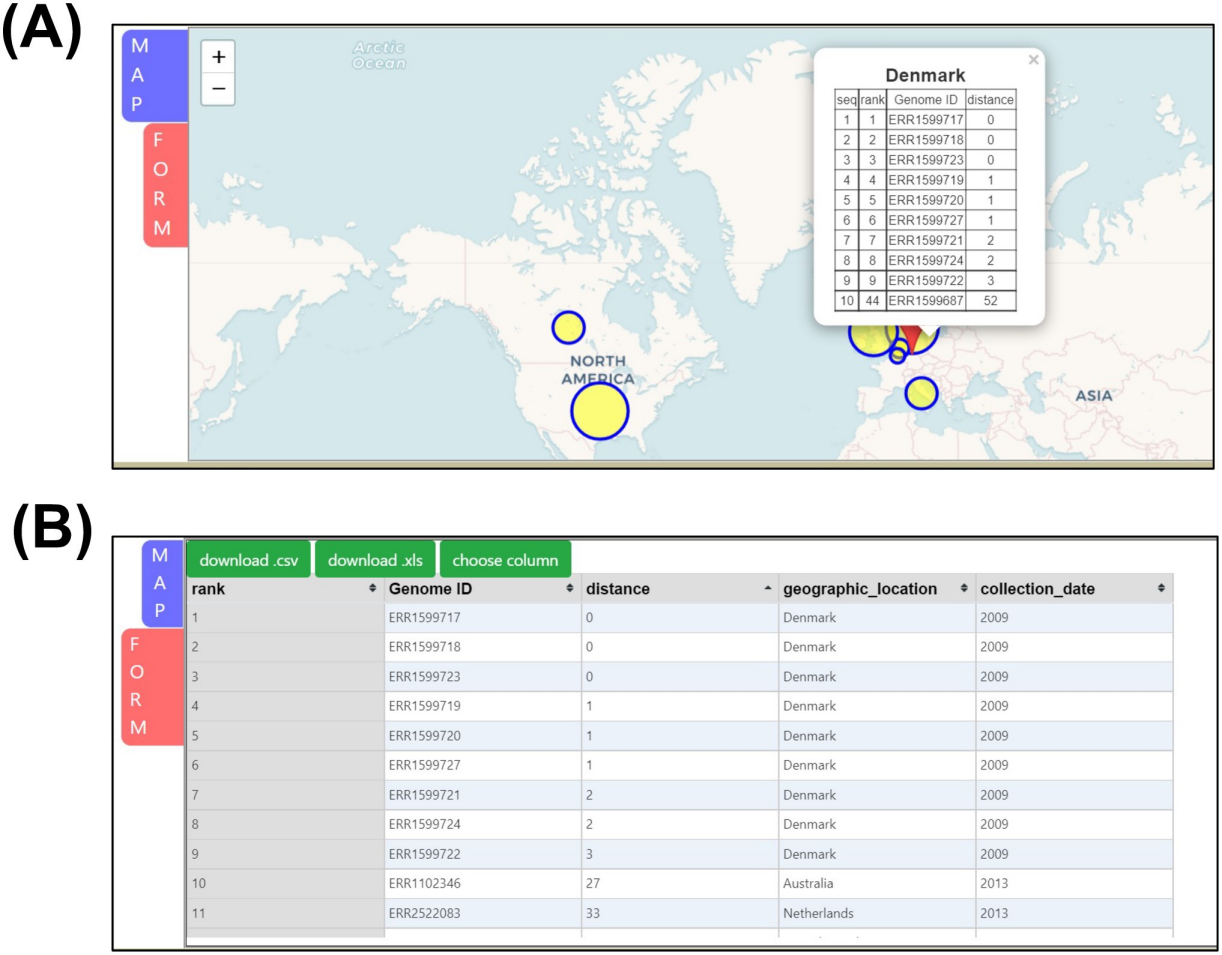
Example analysis. a) list of most similar isolates compared with uploaded isolate presented in LmTraceMap result page MAP tag; b) epidemiological table listing top 50 most similar isolates in result page FORM tag. (Base map and data from OpenStreetMap and OpenStreetMap Foundation. Reprinted from OpenStreetMap under a CC BY license, with permission from OpenStreetMap, original copyright 2020. ©OpenStreetMap contributors. Annotated by the authors).

For demonstration of our web server, we downloaded an *L*. *monocytogenes* genome from NCBI SRA database (Run ID: ERR1599718). This isolate was collected from regular surveillance, and the whole-genome raw reads were submitted by Statens Serum Institute, Denmark in 2016. We assembled the downloaded raw reads by using SPAdes software [32] and submitted them to LmTranceMap. The search results of ERR1599718 are presented in Fig 3. The *MAP* result page is presented in Fig 3(a); this figure demonstrates that 9 isolates in Denmark are the same as the uploaded isolate with the corresponding distance being 0–3 alleles, whereas other isolates have an average distance of 47 alleles. The *FORM* result page is presented in Fig 3(b); this figure indicates that the top rank 1–9 have a distance of 0–3 alleles compared with the uploaded isolate. The geographic locations are all in Denmark, and the collection dates are all concentrated in 2009. On the basis of the query results, we propose that the uploaded isolate might have an epidemiological relationship with ERR1599717, ERR1599718, ERR1599723, ERR1599719, ERR1599720, ERR1599727, ERR1599721, ERR1599724, and ERR1599722. A study published in 2016 in Nature Microbiology [33] confirmed that the epidemiological relationships among the uploaded isolates and the 9 closely related isolated in this study all belong to the same outbreak cluster: *Mom-a*. This confirmation is an example of how to use LmTraceMap to trace all of the possible epidemiological related isolates across distinct regions wordwide.

## Discussion and conclusion

The investigation of foodborne illness clusters is a labor- and time-consuming process. Large-scale comparison among isolates collected from various regions are generally required; therefore, an easy-to-use platform that assists public-health professionals in epidemiological investigations is imperative. Currently, the LmTraceMap system maintains approximately 20 000 *L*. *monocytogenes* epidemiological data items and cgMLST profiles, and backend data are continually updated. Although only an *L*. *monocytogenes* database was created in this study, additional crucial foodborne pathogens could be added in the future.

## Supporting information

S1 Table Number of genomes for each ST type. (XLSX)

S1 Fig Time span of downloaded isolates used in constructing Lm trace database. (PDF)

S2 Fig Predicted 6 serogroups ratio for downloaded isolates used in constructing Lm trace database. (PDF)

S1 File List of Listeria monocytogenes WGS downloaded from NCBI SRA database. (CSV)

S2 File The allele profile used in LmTraceMap database. (ZIP)
