# Supplementary figures and images for: LmTraceMap: A Listeria monocytogenes fast-tracing platform for global surveillance
Source: PLoS One. 2022 May 9;17(5):e0267972. doi: 10.1371/journal.pone.0267972 (PMC9084517; doi:10.1371/journal.pone.0267972)

Figure S1

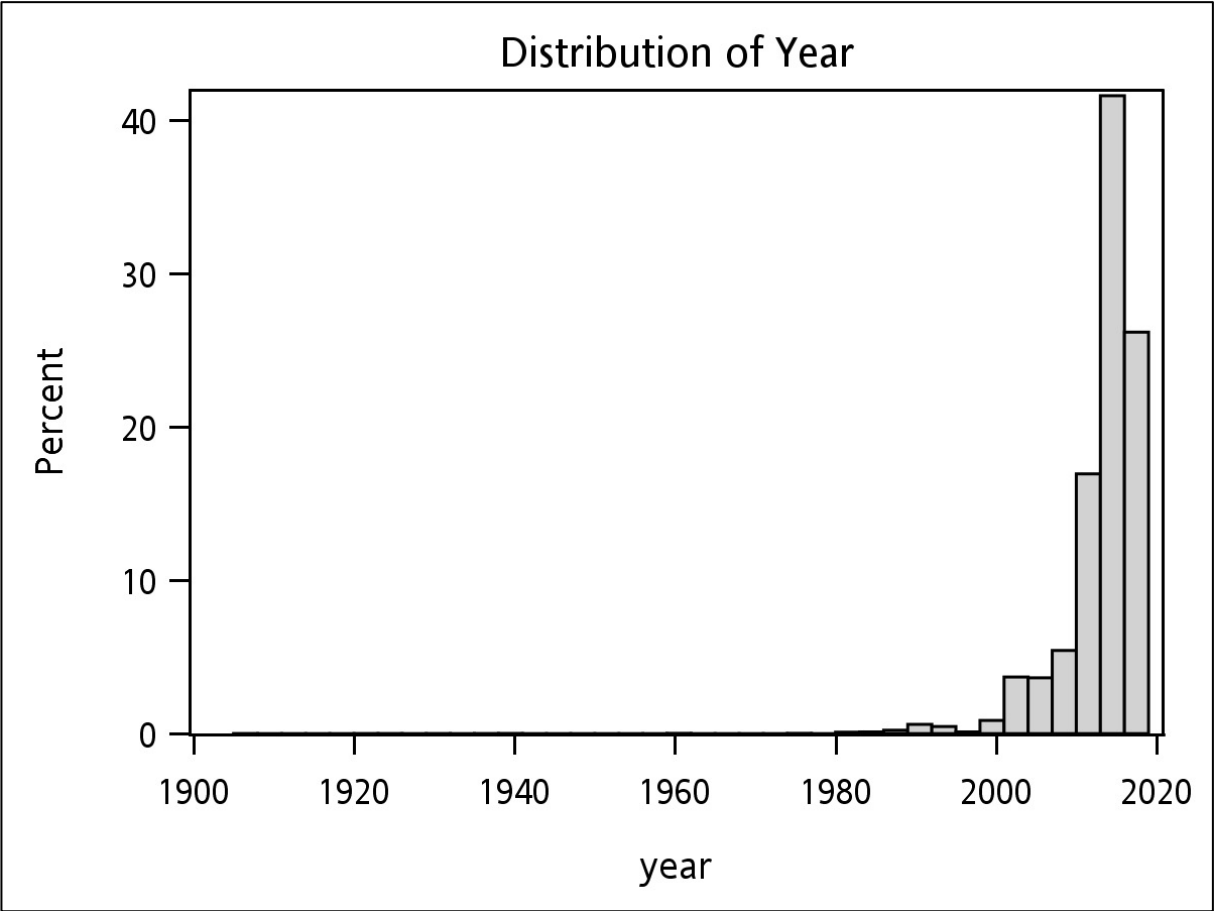

Supplement: S1 Fig — (PDF) [file pone.0267972.s002.pdf]

Figure S2

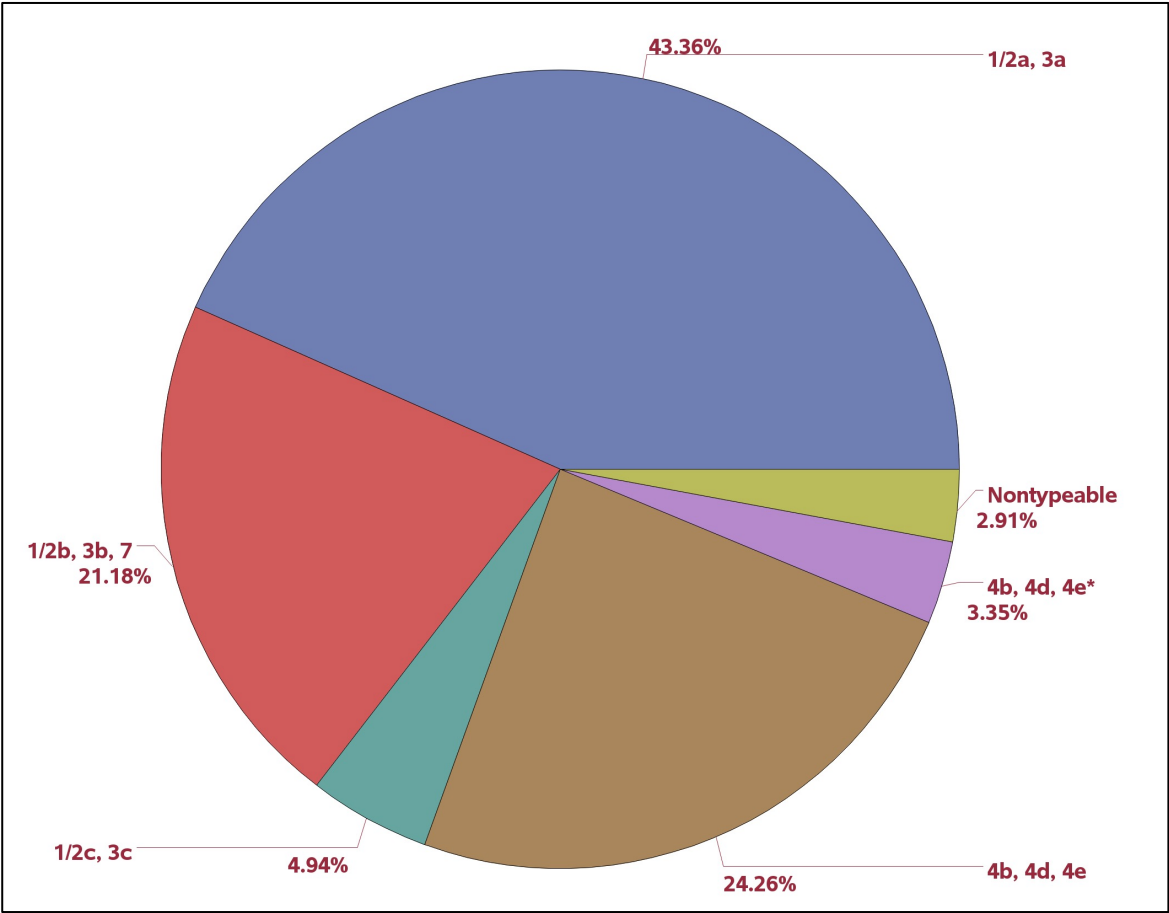

Supplement: S2 Fig — (PDF) [file pone.0267972.s003.pdf]
